# Confirmation of Decreased Rates of Cerebral Protein Synthesis *In Vivo* in a Mouse Model of Tuberous Sclerosis Complex

**DOI:** 10.1523/ENEURO.0480-21.2022

**Published:** 2022-08-01

**Authors:** Rachel Michelle Saré, Anita Torossian, Inna Loutaev, Carolyn Beebe Smith

**Affiliations:** Section on Neuroadaptation and Protein Metabolism, National Institute of Mental Health, National Institutes of Health, Department of Health and Human Services, Bethesda, MD 20814

**Keywords:** mTOR, protein synthesis, tuberous sclerosis

## Abstract

Tuberous sclerosis complex (TSC) is an autosomal dominant disorder that results in intellectual disability and, in ∼50% of patients, autism spectrum disorder. The protein products that are altered in TSC (TSC1 and TSC2) form a complex to inhibit the mammalian target of rapamycin [mTOR; mTOR complex 1 (mTORC1)] pathway. This pathway has been shown to affect the process of mRNA translation through its action on ribosomal protein S6 and 4-elongation binding protein 1. It is thought that mutations in the TSC proteins lead to upregulation of the mTORC1 pathway and consequently an increase in protein synthesis. Unexpectedly, our previous study of a mouse model of TSC (*Tsc2^Djk^*^+/−^) demonstrated decreased *in vivo* rates of protein synthesis throughout the brain. In the present study, we confirm those results in another *Tsc2*^+/−^ mouse model, one with a different mutation locus and on a mixed background (*Tsc2^Mjg^*^+/−^). We also examine mTORC1 signaling and possible effects of prior isoflurane anesthesia. Because measurements of protein synthesis rates *in vivo* require surgical preparation of the animal and anesthesia, we examine mTORC1 signaling pathways both under baseline conditions and following recovery from anesthesia. Our results demonstrate regionally selective effects of prior anesthesia. Overall, our results in both *in vivo* models suggest divergences from the central hypothesis regarding TSC and show the importance of studying protein synthesis *in vivo*.

## Significance Statement

Protein synthesis is an important process for brain function. In the disorder, tuberous sclerosis complex (TSC), the inhibition of the mammalian target of rapamycin (mTOR) pathway is reduced and this is thought to lead to excessive protein synthesis. Most studies of protein synthesis in models of TSC have been conducted *in vitro*. We report here confirmation of our previous *in vivo* study showing decreased brain protein synthesis rates in a second mouse model of TSC, results counter to the central hypothesis regarding TSC. We also explore the possible influence of prior isoflurane exposure on signaling pathways involved in regulation of protein synthesis. This study highlights a novel aspect of TSC and the importance of studying cellular processes *in vivo*.

## Introduction

Tuberous sclerosis complex (TSC) is an autosomal dominant disorder caused by a mutation in either *TSC1* or *TSC2*. The protein products of *TSC1* and *TSC2* form a complex to inhibit the mammalian target of rapamycin complex 1 (mTORC1), a key regulator of cellular energy status and cell growth (Inoki et al., 2003). Increased activity in mTORC1 leads to subsequent activation of products involved in the regulation of cellular protein synthesis: 40S ribosomal protein subunit S6 and eukaryotic translation initiation factor 4E (eIF4E; Avruch et al., 2001; Gingras et al., 2001). Activation of mTORC1 has been demonstrated in many TSC lesions including tubers.

Activation of mTORC1 has been shown to lead to subsequent phosphorylation of the 40S ribosomal protein subunit S6 and activation of the eIF4E. These changes are consistent with activation of mTORC1 leading to an increase in brain protein synthesis. The link between brain protein synthesis and critical processes such as plasticity and learning and memory suggest that such a change in the regulation of protein synthesis could have serious consequences on brain function. An *ex vivo* study of [^35^S]methionine/cysteine metabolic labeling in hippocampal slices from *Tsc2^Djk^*^+/−^ mice indicated decreased incorporation of radiolabel into protein (Auerbach et al., 2011). To address whether these effects occur in the intact brain of the *Tsc2^Djk^*^+/−^ model, we applied the autoradiographic L-[1-^14^C]leucine method which allows for the simultaneous determination of rates of protein synthesis across all regions of brain. Our results showed reduced rates of cerebral protein synthesis (rCPS) throughout the brain (Saré et al., 2018). Our results and those of the *ex vivo* study were contrary to the central dogma of TSC, and we thought it important to repeat these studies in another *Tsc2* heterozygous model. We chose the *Tsc2^Mjg^*^+/−^ mouse model because it is a model on a mixed 129SV/J and C57BL/6J background and it has a different *Tsc2* mutation locus. Although to our knowledge, there have not been systematic studies comparing strains in various phenotypes in models of TSC, strain differences in mice are known to profoundly affect many phenotypes like behavior (Crawley et al., 1997). One difference between these two models is that *Tsc2^Djk^*^+/−^ mice are reported to have learning and memory deficits (Ehninger et al., 2008), whereas *Tsc2^Mjg^*^+/−^ mice were not found to have learning deficits (Reith et al., 2013).

In the present study, we examined the effects of a heterozygous *Tsc2* mutation on rCPS, and we report here that, in this independent study, rCPS were decreased in all 23 areas of the brain examined. We considered a possible influence of the prior surgical preparation under isoflurane anesthesia that animals underwent. We examined signaling pathways involved in regulating protein synthesis, and our results indicate that phosphorylated forms of some signaling proteins are elevated following isoflurane exposure in a region-specific manner. Our study highlights the need to further investigate the role of *Tsc2* on translation in brain *in vivo*.

## Materials and Methods

### Animals

*Tsc2^Mjg^*^+/−^ heterozygous and wild-type (WT) mice (on a mixed C57BL/6 and 129 background) were a gift from J. Moss (Hernandez et al., 2007) and obtained through M. Gambello. All procedures were performed in accordance with the National Institutes of Health *Guidelines on the Care and Use of Animals* and were approved by the National Institute of Mental Health Animal Care and Use Committee. Mice were maintained in a central facility with a standard 12/12 h light/dark cycle with lights on at 6 A.M. At 10 d of age, ear punches were taken for genotyping. Animals were group housed and weaned at 21 d of age.

### rCPS measurement

Between 90 and 105 d of age, males underwent catheterization of a femoral vein and artery under light isoflurane anesthesia. Mice were induced with 5% isoflurane and maintained with 1–5% isoflurane in oxygen. Mice were kept warm during surgery by means of a heating pad. The duration of anesthesia was 60 min. Mice recovered from anesthesia and surgery overnight and were freely moving during recovery and throughout the procedure for measurement of rCPS. Before the experiment, we measured mean arterial blood pressure, hematocrit, and arterial blood glucose concentration to ensure the animals were in a normal physiological condition (Table 1). All rCPS studies were started in the morning between 9 and 11 A.M. The procedure for measurement of rCPS was performed as previously described (Smith et al., 1988; Qin et al., 2005). Briefly, each animal was injected intravenously with 100 µCi/kg L-[1-^14^C]leucine (60 mCi/mmol; Moravek Inc.), and timed arterial blood samples were collected over the next 60 min to determine the time course of [^14^C]leucine specific activity (SA) in arterial plasma. At 60 min, the animal was euthanized by intravenous administration of Beuthanasia-D (a pentobarbital sodium and phenytoin sodium mix; Merck Sharp & Dohme Corp.), brains were removed and frozen on dry ice, and 20 µm sections of brain were prepared with a CM1850 cryostat (Leica) and collected on gelatin-coated slides (FD Neurotechnologies). The slides were fixed in 10% formalin, dried, and exposed to Super RXN film (Fuji Film Corp.) for 42 d along with previously calibrated [^14^C]methylmethacrylate standards. Autoradiograms were digitized with a QImaging digital camera (QImaging) with a pixel size of 11 µm and MCID Elite image processing system (Interfocus Imaging Ltd). Regions of interest (ROIs) were identified by referencing a mouse brain atlas (Paxinos et al., 2001), and the concentration of ^14^C in each ROI was determined by comparing the optical density with the calibration curve built from the standards on the film. rCPS was computed in 23 ROIs by means of the operational equation:

$$R_{i}=\frac{P_{i}^{*}(T)}{\lambda_{i}\int_{0}^{T}\left[\frac{C_{p}^{*}(t)}{C_{p}}\right]dt},$$

in which R_i_ is the rate of leucine incorporation into protein in the tissue (i). P_i_*(T) is the concentration of ^14^C-labeled protein in the tissue (i) at any given time (T) after injection of the tracer. λ_i_ is the fraction of leucine in the precursor pool available for protein synthesis in the tissue (i) derived from the plasma. The remainder, (1 – λ_i_), comes from proteolysis in the tissue (1). λ_i_ was evaluated in WT and *Tsc2^Djk^*^+/−^ mice and published previously (Saré et al., 2018).

**Table 1:** Physiologic variables for mice prior to rCPS studies

Variable	WT (8)	*Tsc2 ^Mjg^*^+/−^ (5)
Age (d)	96 ± 2	95 ± 2
Body weight (g)	41.0 ± 2.0	41.2 ± 3.2
Hematocrit (%)	49.5 ± 1.7	50.2 ± 1.8
Mean arterial blood pressure (mmHg)	112 ± 4	113 ± 2
Arterial blood glucose (mm)	6.3 ± 0.3	6.2 ± 0.6
Arterial plasma leucine (μm)	137 ± 5	127 ± 6

Values are the means ± SEM for the number of mice indicated in parentheses.

### Western blotting

Animals were studied under two conditions: 24 h following isoflurane anesthesia (isoflurane) and without prior anesthesia (control). The isoflurane condition mimicked the conditions used for surgical preparation of animals for rCPS studies. The control condition was to compare with our previously published Western blot studies on the C57BL/6J background (Saré et al., 2018) and to determine whether prior isoflurane exposure might be altering pathways related to regulation of protein synthesis, thus leading to our counterintuitive results. Animals were decapitated and brains were rapidly dissected on ice into cerebellum, frontal cortex, striatum, thalamus, hippocampus, and parietal cortex and placed in preweighed Precellys lysis tubes (Bertin Corporation). All mice were euthanized by decapitation between 10 and 11 A.M.

Tissue was later thawed at 4°C and homogenized with a Precellys homogenizer in ice-cold 5% (weight/volume) tissue protein extraction reagent solution (T-PER; Thermo Scientific) with 1% EDTA (Thermo Scientific) and 1% Halt Protease and Phosphatase inhibitor cocktail (Thermo Scientific). Protein concentrations were determined by a Pierce BCA Protein Assay kit (Thermo Scientific) and 10 µg of extracted protein was loaded per well on a Bio-Rad mini protein stain-free gel (Bio-Rad) for electrophoresis. Protein was then transferred via semi-dry transfer (Bio-Rad) and exposed to primary antibody overnight at 4°C. The membrane was then incubated in secondary antibody (1:10,000 goat anti-rabbit horseradish peroxidase-linked; Bio-Rad) for 1 h at room temperature and exposed to Clarity substrate (Bio-Rad) and visualized for chemiluminescence using a ChemiDoc MP Imager (Bio-Rad). For normalization of Western blottings, we employed the Stain-Free technology (Bio-Rad) to normalize to total protein in the lane.

Primary antibodies were diluted 1:1000 as follows: pAKT (protein kinase B) Ser 473 (Cell Signaling 4060), pAKT Thr 308 (Cell Signaling 4056), pAMPK (5′ adenosine monophosphate-activated protein kinase; Cell Signaling 2535), pCREB (cAMP response element binding protein; Cell Signaling 9198), peIF2α (eukaryotic translation initiation factor 2α; Cell Signaling 3398), pERK (extracellular regulated kinase; Cell Signaling 3370), pGSK3α/β (Glycogen synthase kinase 3a/b; Cell Signaling 9331), pmTOR (mammalian target of rapamycin; Cell Signaling 5536), p-p70S6K (ribosomal protein S6 kinase) Thr389 (Cell Signaling 9234), p-p70S6K Thr421/Ser424 (Cell Signaling 9204), pS6 235/236 (Cell Signaling 2211), pS6 240/244 (240/244), and tuberin (Cell Signaling 4308).

### Statistical analysis

The number of animals to be studied for the rCPS experiments was determined by power analysis based on our published data in *Tsc2^Djk^*^+/−^ mice (Saré et al., 2018) in which we observed an 8–17% coefficient of variation across 18 ROIs and a genotype difference of 18–31%. We hypothesized similar effect sizes and variability in the *Tsc2^Mjg^*^+/−^ mice. Based on these prior data and a difference between the two groups of 20%, we estimated that we could detect changes in rCPS at the *p* ≤ 0.05 level with a statistical power of 95% with four mice per group. rCPS data were analyzed by means of mixed model ANOVA with genotype as a between subject variable and region as a within subject variable. When appropriate, *post hoc* Bonferroni-corrected *t* tests were performed.

Western blotting data were analyzed by means of ANOVA with genotype and treatment as between subject variables and band as a within subject variable for those proteins with more than one band analyzed (e.g. ERK and GSK3). We analyzed phosphorylation site of S6 as a within subject variable. When appropriate, *post hoc* Bonferroni-corrected *t* tests were performed.

Data are presented as means ± SEM. Statistically significant values *p* ≤ 0.05 are denoted with a *, and trending *p* values 0.05 ≤ *p* ≤ 0.10 are presented with a ∼.

## Results

### Tuberin

**Figure 1.**
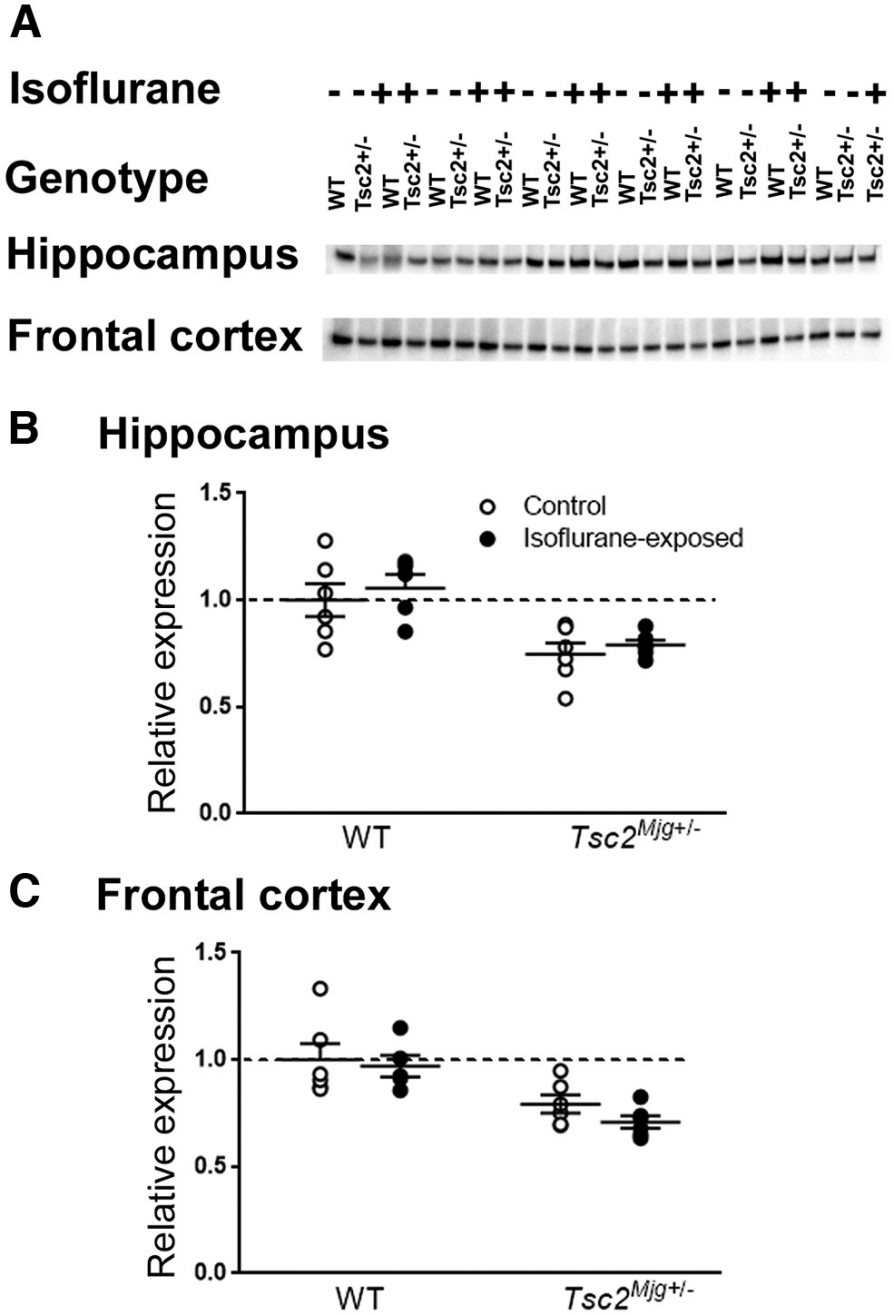
Relative levels of tuberin in WT and *Tsc2^Mjg^*^+/−^ mice in lysates of hippocampus and frontal cortex. Relative levels were measured by means of Western blots (***A***) with two treatments: control and 24 h after a 1-h exposure to isoflurane anesthesia. Data (normalized to WT control mice) were analyzed by means of ANOVA with genotype and treatment as between subject variables. In hippocampus (***B***) neither the genotype × treatment interaction (*F*_(1,19)_ = 0.013, *p* = 0.912) nor the main effect of treatment (*F*_(1,19)_ = 0.758, *p* = 0.395) was statistically significant, but the main effect of genotype (*F*_(1,19)_ = 20.454, *p* < 0.001) was. In frontal cortex (***C***) neither the genotype × treatment interaction (*F*_(1,19)_ = 0.265, *p* = 0.612) nor the main effect of treatment (*F*_(1,19)_ = 1.189, *p* = 0.289) was statistically significant, but the main effect of genotype (*F*_(1,19)_ = 20.115, *p* < 0.001) was. Bars represent the means ± SEM for six mice per group except for the isoflurane-exposed WT which had five mice. Regardless of treatment, tuberin levels were 25% and 24% lower in *Tsc2^Mjg^*^+/−^ mice in hippocampus and frontal cortex, respectively.

We confirmed reduced tuberin (TSC2) in both hippocampus and frontal cortex from *Tsc2^Mjg^*^+/−^ mice. The main effects of genotype were statistically significant in both regions (*p* < 0.001; Fig. 1). Tuberin levels were 25% and 24% lower in *Tsc2^Mjg^*^+/−^ mice in hippocampus and frontal cortex, respectively. Tuberin levels were not affected by prior isoflurane anesthesia.

### rCPS

**Figure 2.**
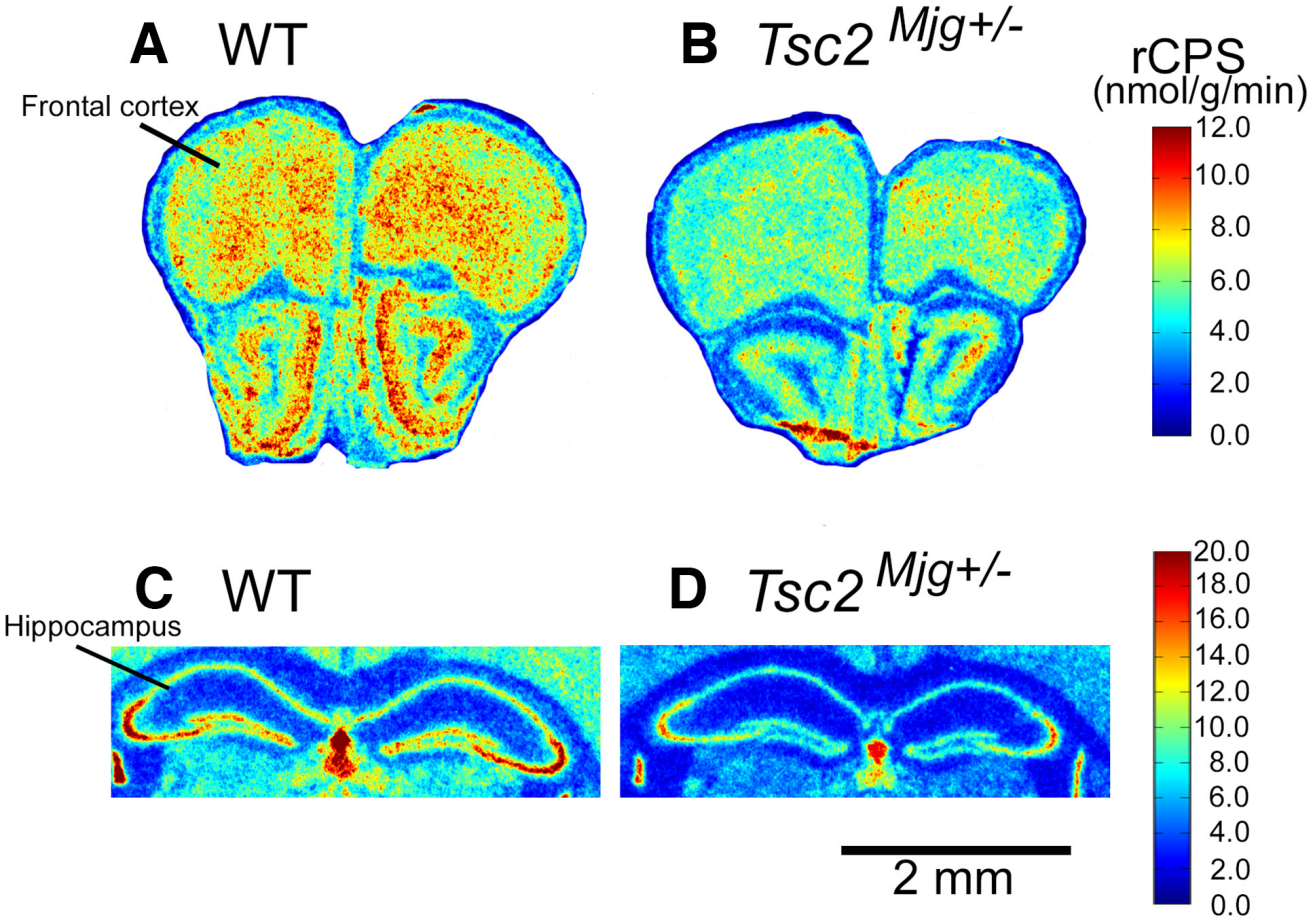
Representative digitized parametric images of rCPS from WT (***A***, ***C***) and *Tsc2^Mjg^*^+/−^ (***B***, ***D***) mice. Images illustrate the patterns of effects of the mutation on rCPS at the level of the frontal association cortex (***A***, ***B***) and dorsal hippocampus (***C***, ***D***). The colorbars on the right define the color scales for the images (upper and lower colorbars pertain to ***A***, ***B*** and ***C***, ***D***, respectively). At both levels, images show that rCPS is decreased in the *Tsc2^Mjg^*^+/−^ mouse compared with WT. Scale bar under ***D*** pertains to all images.

We analyzed rCPS across 23 regions in both WT and *Tsc2^Mjg^*^+/−^ mice and found a statistically significant region × genotype interaction (*F*_(7,75)_ = 9.587; *p* < 0.001). Bonferroni-corrected *post hoc t* tests showed that rCPS in *Tsc2^Mjg^*^+/−^ mice were statistically significantly lower than WT in all 23 regions (*p* ≤ 0.011; Table 2). Differences ranged from 30% in the medial corpus callosum to 60% in the cerebellar flocculus. Representative parametric images at the levels of frontal association cortex and dorsal hippocampus in WT and *Tsc2^Mjg^*^+/−^ mice are illustrated in Figure 2.

**Table 2:** rCPS (nmol/g/min) in WT and *Tsc2 ^Mjg^*^+/−^ **mice**

Region	WT	*Tsc2 ^Mjg^* ^+/−^	Difference (%)	*p* value
Cortex				
Frontal	6.99 ± 0.48 (*n* = 8)	4.82 ± 0.32 (*n* = 5)	−31	0.008
Parietal	8.73 ± 0.50 (*n* = 7)	5.50 ± 0.27 (*n* = 5)	−37	<0.001
Auditory	8.33 ± 0.53 (*n* = 5)	5.22 ± 0.30 (*n* = 5)	−37	<0.001
Visual	8.85 ± 0.75 (*n* = 6)	5.37 ± 0.34 (*n* = 4)	−39	0.001
Corpus callosum				
Medial	2.94 ± 0.14 (*n* = 8)	2.07 ± 0.12 (*n* = 5)	−30	0.001
Lateral	3.31 ± 0.18 (*n* = 8)	2.22 ± 0.14 (*n* = 5)	−33	0.001
Thalamus				
Anterodorsal nucleus	14.67 ± 1.13 (*n* = 5)	8.44 ± 0.86 (*n* = 4)	−42	<0.001
Paraventricular nucleus	13.94 ± 1.59 (*n* = 6)	9.15 ± 0.73 (*n* = 4)	−34	0.011
Dorsomedial	7.65 ± 0.39 (*n* = 6)	4.64 ± 0.40 (*n* = 3)	−39	<0.001
Lateral dorsal nucleus	7.39 ± 0.48 (*n* = 6)	4.50 ± 0.32 (*n* = 4)	−39	<0.001
Medial geniculate nucleus	7.87 ± 0.60 (*n* = 4)	4.21 ± 0.53 (*n* = 5)	−47	<0.001
Ventral posterior	8.37 ± 0.62 (*n* = 8)	5.41 ± 0.36 (*n* = 5)	−35	0.005
Hypothalamus				
Suprachiasmatic nucleus	10.25 ± 0.82 (*n* = 5)	6.96 ± 1.29 (*n* = 4)	−32	0.007
Paraventricular nucleus	15.65 ± 0.85 (*n* = 8)	9.97 ± 1.09 (*n* = 5)	−36	0.002
Supraoptic nucleus	18.09 ± 1.67 (*n* = 6)	9.52 ± 0.90 (*n* = 4)	−47	<0.001
Basolateral amygdala	8.87 ± 0.60 (*n* = 8)	5.66 ± 0.36 (*n* = 5)	−36	0.002
Hippocampus				
Dorsal	7.08 ± 0.39 (*n* = 8)	4.81 ± 0.38 (*n* = 5)	−32	0.002
Ventral	7.02 ± 0.43 (*n* = 6)	4.18 ± 0.27 (*n* = 5)	−41	<0.001
Cerebellum				
Interpeduncular nucleus	9.05 ± 0.62 (*n* = 3)	4.89 ± 0.56 (*n* = 3)	−46	<0.001
Flocculus	9.93 ± 0.88 (*n* = 4)	4.01 ± 0.44 (*n* = 2)	−60	<0.001
Arbor vitae	3.00 ± 0.21 (*n* = 5)	1.32 ± 0.14 (*n* = 4)	−56	<0.001
Simple lobule	10.13 ± 0.86 (*n* = 5)	5.43 ± 0.64 (*n* = 3)	−46	<0.001
Culmen	3.63 ± 0.23 (*n* = 5)	2.25 ± 0.09 (*n* = 3)	−38	<0.001

There were no statistically significant genotype differences.

### Signaling pathways

**Figure 3.**
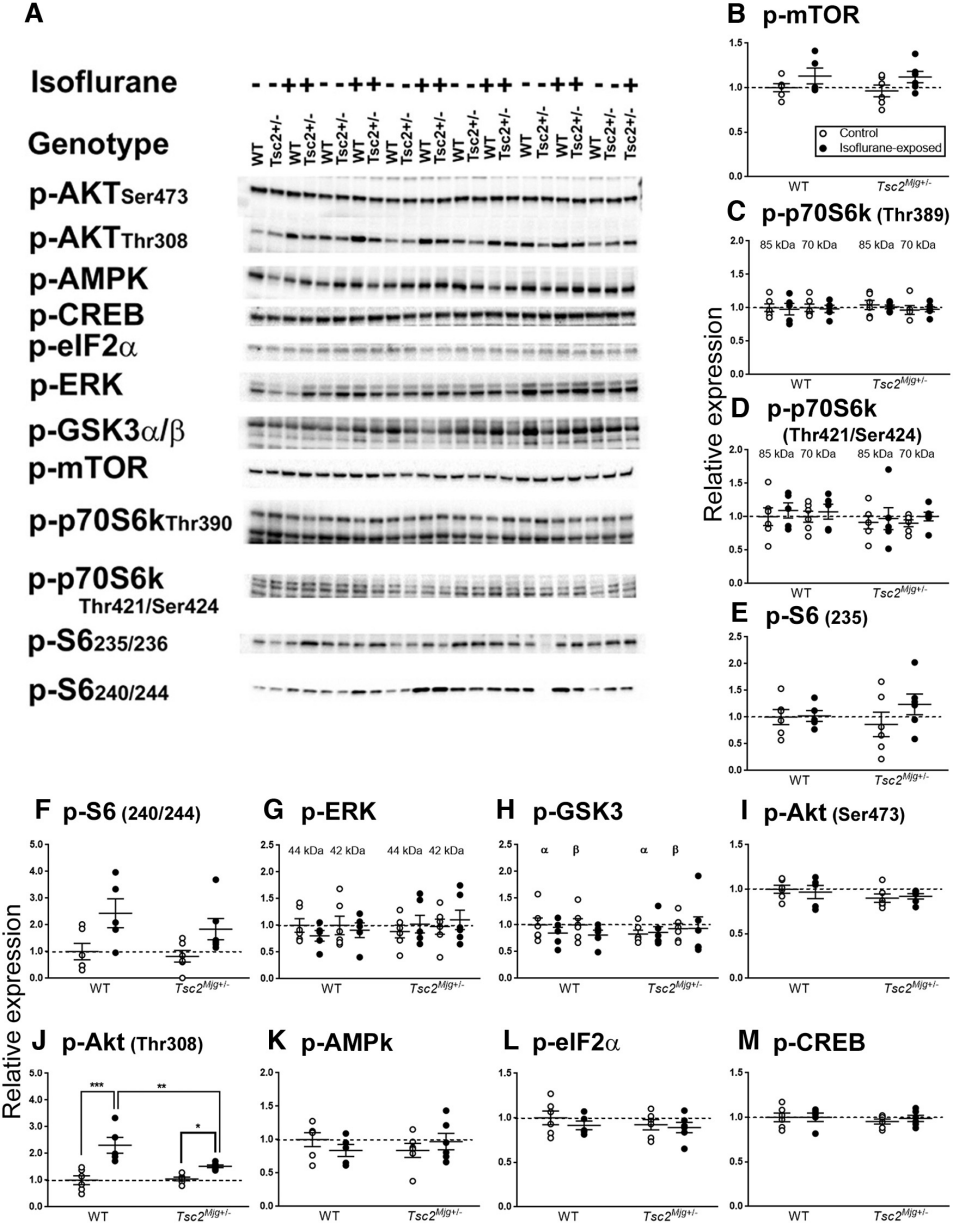
Relative levels of signaling proteins known to affect protein synthesis in lysates of hippocampus from WT and *Tsc2^Mjg^*^+/−^ mice. Relative levels were measured by means of Western blots (***A***) with two treatments: control and 24 h after a 1-h exposure to isoflurane anesthesia. Data were analyzed by means of ANOVA with genotype and treatment as between subject variables (Table 3). Aligned dot plots (***B–M***) indicate protein expression (normalized to WT control mice). Solid horizontal lines represent means ± SEM for six WT control, five WT isoflurane-treated, six *Tsc2^Mjg^*^+/−^ control, and six *Tsc2^Mjg^*^+/−^ isoflurane-treated mice. Horizontal dashed line represents a relative expression of 1.0. For Akt Thr 308 (***J***) the genotype × treatment interaction was statistically significant and results of *post hoc* Bonferroni corrected *t* tests are shown on the figure; *0.01 ≤ *p* ≤ 0.05, **0.001 ≤ *p* ≤ 0.01, ***p ≤ 0.001.

**Figure 4.**
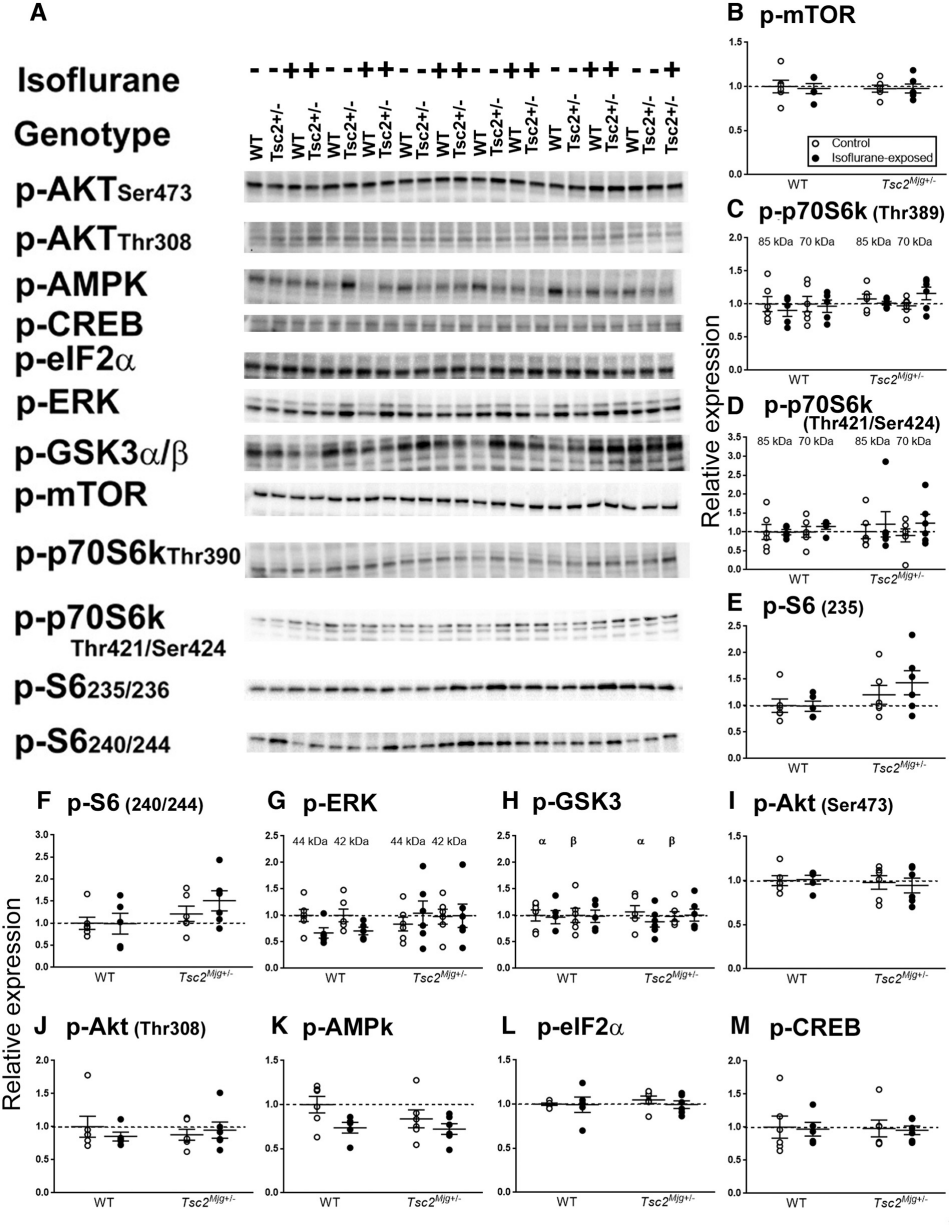
Relative levels of signaling proteins known to affect protein synthesis in lysates of frontal cortex from WT and *Tsc2^Mjg^*^+/−^ mice. Relative levels were measured by means of Western blots (***A***) with two treatments: control and 24 h after a 1-h exposure to isoflurane anesthesia. Data were analyzed by means of ANOVA with genotype and treatment as between subject variables (Table 3). Aligned dot plots (***B–M***) indicate protein expression (normalized to WT control mice). Solid horizontal lines represent means ± SEM for six WT control, five WT isoflurane-treated, six *Tsc2^Mjg^*^+/−^ control, and six *Tsc2^Mjg^*^+/−^ isoflurane-treated mice. Horizontal dashed line represents a relative expression of 1.0.

Measurements of rCPS were conducted in mice under awake, behaving conditions. To measure rCPS, mice were surgically prepared by insertion of vascular catheters under isoflurane anesthesia. We waited 24 h after surgery/anesthesia to measure rCPS to ensure that animals of both genotypes were in a normal physiological state. We considered prior use of isoflurane anesthesia and possible differential effects on the two genotypes as a potential confounding factor. Whereas it was impossible to directly test the effects of prior anesthesia on rCPS, as a surrogate we tested possible effects on signaling pathways known to affect protein synthesis. We analyzed the phosphorylated forms of 12 proteins in two regions of brain, hippocampus (Fig. 3) and frontal cortex (Fig. 4). We compared two treatments: (1) control (unexposed to isoflurane) and (2) isoflurane (24 h following 60 min of isoflurane exposure), in groups of WT and *Tsc2^Mjg^*^+/−^ mice.

**Table 3:** ANOVA results of Western blots of hippocampus

Protein	Interaction	Main effect	*F*_(df,error)_ value	*p* value
pAKT Ser473	Treatment × Genotype		*F*_(1,19)_ = 0.190	0.668
		Treatment	*F*_(1,19)_ = 0.097	0.759
		Genotype	*F*_(1,19)_ = 1.820	0.193
pAKT Thr308	Treatment × Genotype		*F*_(1,19)_ = 6.766	0.018^*^
		Treatment	*F*_(1,19)_ = 30.807	<0.001^*^
		Genotype	*F*_(1,19)_ = 5.334	0.032^*^
pAMPK	Treatment × Genotype		*F*_(1,19)_ = 1.856	0.189
		Treatment	*F*_(1,19)_ = 0.018	0.895
		Genotype	*F*_(1,19)_ = 0.019	0.893
pCREB	Treatment × Genotype		*F*_(1,19)_ = 0.170	0.684
		Treatment	*F*_(1,19)_ = 0.195	0.663
		Genotype	*F*_(1,19)_ = 0.540	0.471
peIF2α	Treatment × Genotype		*F*_(1,19)_ = 0.177	0.678
		Treatment	*F*_(1,19)_ = 0.837	0.372
		Genotype	*F*_(1,19)_ = 0.651	0.430
pERK	Treatment × Genotype × Band		*F*_(1,19)_ = 0.155	0.699
	Treatment × Band		*F*_(1,19)_ = 0.069	0.796
	Genotype × Band		*F*_(1,19)_ = 0.328	0.574
	Treatment × Genotype		*F*_(1,19)_ = 0.652	0.429
		Treatment	*F*_(1,19)_ = 0.001	0.971
		Genotype	*F*_(1,19)_ = 0.248	0.624
		Band	*F*_(1,19)_ = 127.210	<0.001^*^
pGSK3α/β	Treatment × Genotype × Band		*F*_(1,19)_ = 0.661	0.426
	Treatment × Band		*F*_(1,19)_ = 0.227	0.639
	Genotype × Band		*F*_(1,19)_ = 1.259	0.276
	Treatment × Genotype		*F*_(1,19)_ = 0.692	0.416
		Treatment	*F*_(1,19)_ = 0.388	0.541
		Genotype	*F*_(1,19)_ = 0.262	0.614
		Band	*F*_(1,19)_ = 265.256	<0.001^*^
pmTOR	Treatment × Genotype		*F*_(1,19)_ = 0.032	0.861
		Treatment	*F*_(1,19)_ = 4.789	0.041^*^
		Genotype	*F*_(1,19)_ = 0.135	0.717
p-p70 S6K Thr389	Treatment × Genotype × Band		*F*_(1,19)_ = 0.191	0.667
	Treatment × Band		*F*_(1,19)_ = 0.096	0.760
	Genotype × Band		*F*_(1,19)_ = 1.127	0.302
	Treatment × Genotype		*F*_(1,19)_ = 0.020	0.889
		Treatment	*F*_(1,19)_ = 0.046	0.832
		Genotype	*F*_(1,19)_ = 0.003	0.955
		Band	*F*_(1,19)_ = 325.069	<0.001^*^
p-p70 S6K Thr421/Ser424	Treatment × Genotype × Band		*F*_(1,19)_ = 0.231	0.636
	Treatment × Band		*F*_(1,19)_ = 0.463	0.504
	Genotype × Band		*F*_(1,19)_ = 0.075	0.788
	Treatment × Genotype		*F*_(1,19)_ <0.001	0.998
		Treatment	*F*_(1,19)_ = 0.762	0.394
		Genotype	*F*_(1,19)_ = 0.942	0.344
		Band	*F*_(1,19)_ = 140.106	<0.001^*^
pS6	Treatment × Genotype × Site		*F*_(1,19)_ = 1.343	0.261
	Treatment × Site		*F*_(1,19)_ = 0.406	0.532
	Genotype × Site		*F*_(1,19)_ = 0.506	0.485
	Treatment × Genotype		*F*_(1,19)_ = 0.337	0.569
		Treatment	*F*_(1,19)_ = 6.396	0.020^*^
		Genotype	*F*_(1,19)_ = 0.092	0.765
		Site	*F*_(1,19)_ = 38.962	<0.001^*^

Values are the mean ± SEM for the number of mice indicated in parentheses. Whereas experiments were completed in eight WT and five *Tsc2^Mjg^*^+/−^ mice, the number of mice analyzed depended on the quality of the autoradiograms at the level of each ROI. Bonferroni-corrected *post hoc* tests showed that *Tsc2^Mjg^*^+/−^ mice had statistically significantly lower rCPS in all brain regions analyzed.

### Hippocampus

For pmTOR the main effect of treatment was statistically significant (*p* = 0.041) indicating an increase in pmTOR regardless of genotype 24 h following exposure to isoflurane (Table 3; Fig. 3). Similarly, for pS6 the main effect of treatment was statistically significant (*p* = 0.020; Table 3), showing increased pS6, regardless of phosphorylation site or genotype, following isoflurane exposure. For pAKT Thr308 the genotype × treatment interaction was statistically significant (*p* = 0.018; Table 3) indicating that the effects of isoflurane treatment in the two genotypes differed. *Post hoc t* tests showed that whereas in both genotypes pAKT Thr308 expression was increased following isoflurane exposure, the response was greater in WT mice (130% increase, *p* < 0.001) compared with *Tsc2^Mjg^*^+/−^ mice (45% increase, *p* = 0.046). Other signaling proteins were not statistically significantly affected.

### Frontal cortex

In frontal cortex, we did not find these effects on pmTOR, pS6, and pAkt Thr308 (Table 4). For pS6 we found a statistically significant (*p* = 0.022) main effect of genotype, indicating that regardless of phosphorylation site, pS6 was higher in *Tsc2^Mjg^*^+/−^ mice compared with WT mice. For pAMPK the main effect of treatment was statistically significant (*p* = 0.035; Table 4). The phosphorylated form of AMPK was lower following exposure to isoflurane regardless of genotype (Fig. 4). There was also a treatment by band interaction for p-p70 S6K Thr389 (*p* = 0.048; Table 4), suggesting a differential reaction for each band of p=p70 S6K following isoflurane treatment (Fig. 4).

**Table 4:** ANOVA results of Western blots of frontal cortex

Protein	Interaction	Main effect	*F*_(df,error)_ value	*p* value
pAKT Ser473	Treatment × Genotype		*F*_(1,19)_ = 0.106	0.749
		Treatment	*F*_(1,19)_ = 0.030	0.865
		Genotype	*F*_(1,19)_ = 0.378	0.546
pAKT Thr308	Treatment × Genotype		*F*_(1,19)_ = 0.851	0.368
		Treatment	*F*_(1,19)_ = 0.109	0.745
		Genotype	*F*_(1,19)_ = 0.012	0.913
pAMPK	Treatment × Genotype		*F*_(1,19)_ = 0.765	0.393
		Treatment	*F*_(1,19)_ = 5.166	0.035^*^
		Genotype	*F*_(1,19)_ = 1.115	0.304
pCREB	Treatment × Genotype		*F*_(1,19)_ = 0.043	0.838
		Treatment	*F*_(1,19)_ = 2.655	0.120
		Genotype	*F*_(1,19)_ = 0.033	0.859
peIF2α	Treatment × Genotype		*F*_(1,19)_ = 0.235	0.634
		Treatment	*F*_(1,19)_ = 0.370	0.550
		Genotype	*F*_(1,19)_ = 0.265	0.613
pERK	Treatment × Genotype × Band		*F*_(1,19)_ = 1.174	0.292
	Treatment × Band		*F*_(1,19)_ = 0.560	0.464
	Genotype × Band		*F*_(1,19)_ = 0.303	0.588
	Treatment × Genotype		*F*_(1,19)_ = 2.099	0.164
		Treatment	*F*_(1,19)_ = 0.350	0.561
		Genotype	*F*_(1,19)_ = 0.402	0.533
		Band	*F*_(1,19)_ = 145.346	<0.001^*^
pGSK3α/β	Treatment × Genotype × Band		*F*_(1,19)_ = 0.971	0.337
	Treatment × Band		*F*_(1,19)_ = 1.758	0.201
	Genotype × Band		*F*_(1,19)_ = 0.013	0.911
	Treatment × Genotype		*F*_(1,19)_ = 0.240	0.630
		Treatment	*F*_(1,19)_ = 0.568	0.460
		Genotype	*F*_(1,19)_ = 0.004	0.953
		Band	*F*_(1,19)_ = 260.590	<0.001^*^
pmTOR	Treatment × Genotype		*F*_(1,19)_ = 0.052	0.822
		Treatment	*F*_(1,19)_ = 0.034	0.856
		Genotype	*F*_(1,19)_ = 0.046	0.832
p-p70 S6K Thr389	Treatment × Genotype × Band		*F*_(1,19)_ = 2.292	0.146
	Treatment × Band		*F*_(1,19)_ = 4.451	0.048^*^
	Genotype × Band		*F*_(1,19)_ = 0.001	0.975
	Treatment × Genotype		*F*_(1,19)_ = 0.882	0.359
		Treatment	*F*_(1,19)_ = 0.090	0.767
		Genotype	*F*_(1,19)_ = 1.228	0.282
		Band	*F*_(1,19)_ = 180.660	<0.001^*^
p-p70 S6K Thr421/Ser424	Treatment × Genotype × Band		*F*_(1,19)_ = 0.085	0.773
	Treatment × Band		*F*_(1,19)_ = 0.003	0.957
	Genotype × Band		*F*_(1,19)_ = 0.279	0.603
	Treatment × Genotype		*F*_(1,19)_ = 0.265	0.613
		Treatment	*F*_(1,19)_ = 0.535	0.473
		Genotype	*F*_(1,19)_ = 0.150	0.702
		Band	*F*_(1,19)_ = 30.355	<0.001^*^
pS6	Treatment × Genotype × Site		*F*_(1,19)_ < 0.001	0.985
	Treatment × Site		F_(1,19)_ < 0.001	0.993
	Genotype × Site		*F*_(1,19)_ = 0.049	0.826
	Treatment × Genotype		*F*_(1,19)_ = 0.947	0.343
		Treatment	*F*_(1,19)_ = 0.813	0.378
		Genotype	*F*_(1,19)_ = 6.216	0.022^*^
		Site	*F*_(1,19)_ = 6.634	0.019^*^

* Denotes statistical significance *p* ≤ 0.05.

## Discussion

We measured regional rCPS *in vivo* and found that rCPS were statistically significantly lower in adult *Tsc2^Mjg^*^+/−^ mice compared with WT in all 23 brain regions examined. This present result confirms our previous study in a different mouse model of TSC (Saré et al., 2018). We considered other possible explanations for these counterintuitive results. In both studies, animals had undergone surgical implantation of catheters under isoflurane anesthesia. Despite the 24-h recovery time, the prior surgical preparation under isoflurane anesthesia used in both studies may have affected rCPS measurements differentially in the two genotypes. Whereas we could not directly test this hypothesis, we did measure levels of signaling molecules that have known effects on mRNA translation. Our results in hippocampus indicate that prior treatment with isoflurane may increase protein synthesis via the mTORC1 pathway in both WT and *Tsc2^Mjg^*^+/−^ mice, and that effects as indicated by the phosphorylation of Akt (Thr 308) were considerably greater in WT. Similar effects were not evident in frontal cortex in which prior isoflurane exposure resulted in decreased pAMPK.

Our results are an important confirmation that rCPS is in fact decreased in *Tsc2*^+/−^ compared with WT mice (Saré et al., 2018). Although this is surprising in light of the known literature regarding mTORC1, it is important to note that most of the research on mTORC1 was conducted in cell lines and focused on specific signaling molecules, whereas we measured rCPS *in vivo* and looked at the global process of translation. Results of our *in vivo* studies suggest that mTORC1 regulation *in vivo* is more complicated than previously appreciated and that there are likely compensatory changes through feedback loops modulating these changes (Huang and Manning, 2009).

The direction of changes in rCPS is the same as seen previously (Saré et al., 2018), but measured rCPS values in both WT and *Tsc2^Mjg^*^+/−^ were higher in the present study. Interestingly, we noted that the mice used in this study (WT and *Tsc2^Mjg^*^+/−^), were morbidly obese (*p* < 0.001) with significantly higher leucine values (*p* < 0.001; 16% increase) but similar mean arterial blood pressures and plasma glucose concentrations (Table 5). Moreover, in the present study we used values of λ determined in the previous study, and it is possible that values of λ may be altered by the obesity. We also considered the possibility that rCPS values were affected by circadian time and sleep duration in the two genotypes. Experiments in both the present study and our previous study (Saré et al., 2018) were done at the same time of day (between 9 and 11 A.M.) and mice were maintained in the same animal facility with a 12/12 h light/dark cycle with lights on at 6 A.M. Moreover, we have reported that sleep duration in *Tsc2*^+/−^ mice is similar to WT (Saré et al., 2020).

**Table 5:** ANOVA results strain differences in physiological variables

Variable	Interaction	Main effect	*F*_(df,error)_ value	*p* value
Body weight	Strain × Genotype		*F*_(1,30)_ = 0.013	0.911
		Strain	*F*_(1,30)_ = 103.935	<0.001*
		Genotype	*F*_(1,30)_ < 0.001	0.990
Arterial blood pressure	Strain × Genotype		*F*_(1,30)_ = 0.001	0.981
		Strain	*F*_(1,30)_ = 2.300	0.140
		Genotype	*F*_(1,30)_ = 0.065	0.801
Arterial blood glucose concentration	Strain × Genotype		*F*_(1,30)_ = 0.065	0.801
		Strain	*F*_(1,30)_ < 0.001	0.983
		Genotype	*F*_(1,30)_ = 0.0.439	0.513
Arterial plasma leucine concentration	Strain × Genotype		*F*_(1,30)_ = 0.428	0.518
		Strain	*F*_(1,30)_ = 14.494	<0.001*
		Genotype	*F*_(1,30)_ = 2.187	0.150

*Denotes statistical significance *p* ≤ 0.05.

Comparison of physiological variables between the *Tsc2^Mjg^*^+/−^ mice used in this study (values in Table 1) and *Tsc2^Djk^*^+/−^ mice used in our previous study (Saré et al., 2018). Mean values in control and *Tsc2^Djk^*^+/−^ mice, respectively, as follows: body weight: 98 ± 1 and 96 ± 2 g; arterial blood pressure: 109 ± 1 and 109 ± 2 mmHg; arterial blood glucose: 6.5 ± 0.4 and 6.1 ± 0.5 mm; arterial plasma leucine: 116 ± 4 and 111 ± 3 μm.

Our study of phosphorylated forms of select signaling proteins indicate increased pS6 in frontal cortex but not in hippocampus in *Tsc2^Mjg^*^+/−^ mice and decreased pAktThr308 in hippocampus of *Tsc2^Mjg^*^+/−^ mice but not in frontal cortex. The regional difference is surprising since both regions had similar decreases in tuberin (∼25%) and in rCPS. To our knowledge, there are no other reports of regionally differential effects on the mTORC1 pathway in a TSC mouse model. In other mouse models of TSC, reported effects were similar in both cortex and hippocampus (Way et al., 2009; Magri et al., 2011; Koene et al., 2019). These regional differences in our study highlight the complexity of signaling pathways and feedback loops *in vivo*.

In addition to genotype-specific changes in phosphorylation of signaling proteins, we also found condition-specific effects. Prior treatment with isoflurane increased pmTOR, pS6 and pAktThr308 in hippocampus but not in frontal cortex in both genotypes. These changes extend the results of other studies of effects of isoflurane and halothane (another halogenated ether formerly used for anesthesia) on phosphorylated signaling proteins (Palmer et al., 2006; Antila et al., 2017; Leikas et al., 2017; Zhang et al., 2019). In adult rats 20-min exposure to isoflurane resulted in increased pAkt Thr308 and pGSK3β in both cortex and striatum (Leikas et al., 2017). Studies of the effects of halothane anesthesia on perfused rat liver indicate that halothane decreased rates of protein synthesis in a dose-dependent and time-dependent manner (Palmer et al., 2006). These effects were accompanied by an increase in peIF2α and decreases in pS6 and pP70S6k, consistent with the decreased protein synthesis rates. Another study reported effects of 30 min isoflurane exposure on behavior and phosphorylation of signaling proteins in rodents (Antila et al., 2017). In WT mice, 30 min of isoflurane exposure resulted in increased pAkt Thr308, pmTOR and pP70S6k in prefrontal cortex and increased pP70S6k but no change in pAkt Thr308 or pmTOR in hippocampus; these effects were seen immediately following isoflurane exposure (Antila et al., 2017). Some behavioral effects were seen days after the isoflurane exposure. Taken together, Western blot results indicate acute effects of isoflurane exposure differ from effects seen after a 24-h recovery, and effects are regionally specific. Clearly there is no simple accounting for rCPS effects by analysis of these select signaling proteins, but the results do indicate both genotype and condition (prior isoflurane) effects. Regional differences may reflect the heterogeneity of brain in terms of cell types, density of synaptic terminals and predominant neurotransmitter. It would be interesting to measure rCPS in animals without the use of isoflurane or with longer recovery times, but surgical insertion of vascular catheters is essential for the method. Future studies should determine the time course of signaling changes following isoflurane exposure with the aim of finding an optimal recovery time for measurement of rCPS.

In summary, we found in multiple studies, that *Tsc2* heterozygous deficiency results in reduced regional rCPS. Although we observed increased pS6 (indicative of activated mTORC1) in the frontal cortex, and an effect of prior isoflurane administration in the hippocampus, these local results are unlikely to explain the global changes in rCPS. Our results highlight the importance of understanding the complexity of the mTORC1 pathway regulation *in vivo.*

## Synthesis

Reviewing Editor: Lynda Erskine, University of Aberdeen Decisions are customarily a result of the Reviewing Editor and the peer reviewers coming together and discussing their recommendations until a consensus is reached. When revisions are invited, a fact-based synthesis statement explaining their decision and outlining what is needed to prepare a revision will be listed below. The following reviewer(s) agreed to reveal their identity: Benjamin Auerbach, Tomi Rantamaki.

Thank you for submitting your manuscript to eNeuro which has now been reviewed by 2 experts in the field. The reviewers can see merit in the publication of this confirmatory study. However, they have a number of concerns about the framing and rigour of the experiments. Most importantly, is how sample sizes were justified as the n varies from brain region to brain region for the rCPS measurements. Substantial revisions, including potentially new experiments, will be required before the paper would be suitable for publication.

Below is a synthesis of the reviewers’ comments, including the list of issues we are all agreed need to be addressed.

General comments/concerns raised by the reviewers’:

This paper uses autoradiogaphic L-[1-14C]leucine to make in vivo measurements of bulk cerebral protein synthesis rates (rCPS) in a Tsc2Mjg+/- mouse model of Tuberous Sclerosis Complex (TSC). In addition, the authors examine levels of phosphorylated signaling proteins involved in protein synthesis regulation in animals with and without prior isoflurane exposure to consider the influence of anesthesia, which was required for surgical preparation for rCPS measurements. The authors confirmed their previous findings that Tsc2Mjg+/- mice exhibit reduced rCPS across brain regions in much the same manner as Tsc2Djk+/- mice, which are on a different background strain (Tsc2Mjg+/- : mixed 129SV/J and c57BL/6J vs Tsc2Djk +/-: c57BL/6J) and have a different Tsc2 mutation locus (although both mutations result in a premature stop codon and ∼25% decrease in Tuberin protein levels). They did not find any systematic effect of isoflurane on protein phosphorylation levels, suggesting that anesthesia exposure does not confound interpretation of the rCPS measurements. While it is commendable that the authors have replicated their results in a different background strain, the difference between the model used here and in previous studies appear to be rather minimal and thus the impact of these findings are somewhat incremental. Coupled with the relatively sparse introduction and discussion sections, it was difficult to determine the overall rationale and implications of this study. Additional experiments or at the very least a more thorough discussion of why these experiments are important and how the results move the field forward are required to consider this manuscript for publication.

Major points to be addressed:

1. Replication of previous findings in a distinct mouse model of TSC (albeit with a functionally very similar mutation) is important, but it feels like there are some missed opportunities here. What are the differences between strain used here and in previous studies and is there reason to believe there would be differences in metabolic rates between these two mouse strains? What type of strain-specific effects been seen for other phenotypes in TSC models (or other related neurodevelopmental disorders), that would indicate that background strain may be influencing disease phenotypes? Most importantly, what about other TSC models that differ from Tsc2Djk +/- mice in more substantial ways?

There are many mouse models of TSC with distinct types of mutations, expression patterns, etc, where this type of comparison would be more informative. In particular, a major question to be resolved is if more severe depletion of Tsc1 or 2 levels (e.g. beyond the 25% reduction seen in Tsc2+/- animals) has different effects on mTOR signaling and protein synthesis rates in the intact brain. For instance, a recent study found that mice with one null Tsc2 allele and one conditional hypomorphic Tsc2 allele (Tsc2k/c-del3) in neurons, resulting in >90% decrease in tuberin levels, actually display increased protein synthesis rates using in vitro measurements (Kelly et al., Neuropsychopharm, 2017). It would be very interesting to see if this mouse model exhibits enhanced or decreased cerebral protein synthesis rates in vivo. The difference between heterozygous Tsc1/2 mutation and more complete loss has important implications for our understanding of TSC pathophysiology, as many TSC lesions (including cortical tubers) may be the result of a loss of heterozygosity or “second hit” mutations in cells with only one functional Tsc1 or 2 allele.

2. Group sizes are poorly described. Why do N numbers differ so much for rCPS analysis? Were power calculations performed? In some analysis the N number is very low (even N=2) (see e.g. Table 2). It seems odd to have so variable N number between the groups, often more in WTs.

3. Please show specific group values (+/- SEMs) for the data presented in Table 5.

4. The author suggest that reduced rCSP in Tsc2+/- animals may be due to compensatory changes, due in part to the fact that they did not observe changes in mTOR activity or downstream signaling components. A crucial experiment to test this hypothesis would be to determine if altered rCSP in Tsc2+/- animals is sensitive to acute manipulation of mTOR activity. In a previous study, it was shown that acute rapamycin treatment ex vivo could ameliorate deficits in protein synthesis rates and protein-synthesis dependent plasticity in hippocampal slices (Auerbach, Osterweil, & Bear, Nature, 2011). Can systemic (or intracerebral) rapamycin treatment reverse the protein synthesis deficits observed in vivo in these animals?

5. For Figures 2 and 3, there is no quantification of total protein levels provided for the signaling proteins examined, only quantification of phosphorylated levels. Since phosphorylation levels will be proportional to total amount of protein, it is difficult to interpret any difference in phosphorylation level without knowing if there is a difference in total protein expression. This is especially important considering the main result of the study is that there is a difference in total protein synthesis rates.

6. The presentation of the western blot data could be improved. Please increase the size of the blots and include estimated molecular weights. Perhaps put representative blots in main figs and full blots to supplements. Include significances in the figures. Perhaps change the order the data is presented. The main reason to conduct WBs was probably to check how the data fit with rCPS? Why not show first WT and Tsc mutants that undergo preceding anesthesia next to one another and then the rest next to one and other?

Western blot data must be normalized (respected total proteins or at least “housekeeping” protein)

7. Cerebral protein synthesis is influenced by a number of factors. Did the authors check e.g. circadian/sleep and activity aspects in the animals?

8. The authors claim they have investigated rCPS in vivo. While the administration of the radioactive leucine is done systemically and hence in vivo, the actual analysis is done ex vivo using brain section.

9. Details of anesthesia are missing: dosing (and flow rates), depth, duration (60 minutes?), temperature control. Sham anesthesia (for the second study)?

10. Altogether the changes in phosphoproteins are minor. This appears very logical given the physiological role of protein phosphorylation (transient). Does anesthesia generally produces transient protein phosphorylation changes? The authors could check the literature more thoroughly (only one cited).

Additional points:

(i) It was unclear why a repeated measures ANOVA was used for S6 phosphorylation

(ii) Were the animals immobilized somehow during i.v. injection? Please also clearly state the time of the day of i.v. injection.

(iii) Mention specific drugs included in Beuthanasia

(iv) How is “frontal cortex” defined here? Could the authors show schematic pic of the dissection?

(v) Table 2 provides a good quantification of regional cerebral protein synthesis rates in WT and Tsc2Mjg+/-animals but a figure with representative autoradiographs should also be included for visual comparison.

(vi) Figure 2+3: It is great to be able to see the effects of anesthesia on signaling cascades in both WT and Tsc2 animals, but with so many bars it is difficult to directly compare WT vs Tsc2 conditions. Since all protein expression levels are normalized WT controls, adding a thin dashed line across the bar graphs at 1.0 would be helpful for visual comparison between genotypes and conditions.

(vii) Anesthesia (grey)/no anesthesia labels are missing in some figs.

(viii) Line 147. “Measurements of rCPS were conducted in mice under awake...” can´t be right. The analysis was done using ex vivo sections.

(ix) While the paper is generally well-written and clear, the introduction and discussion sections are rather sparse and it is difficult to understand the overall goals of the study (beyond replication of previous results) and implications of the results. The authors could do a better job of framing the narrative to make it more impactful.

(x) Line 202. Were WT mice also morbidly obese “(WT and Tsc)”?

(xi) Why are not all the phosphorylation residues mentioned? Please also discuss what these changes mean.
